# Clarifying the Relationships between Microsporidia and Cryptomycota

**DOI:** 10.1111/jeu.12519

**Published:** 2018-04-28

**Authors:** David Bass, Lucas Czech, Bryony A. P. Williams, Cédric Berney, Micah Dunthorn, Frederic Mahé, Guifré Torruella, Grant D. Stentiford, Tom A. Williams

**Affiliations:** ^1^ Pathology and Microbial Systematics Theme Centre for Environment, Fisheries and Aquaculture Science (Cefas) Barrack Road, The Nothe Weymouth DT4 8UB UK; ^2^ Department of Life Sciences The Natural History Museum London SW7 5BD UK; ^3^ Heidelberg Institute for Theoretical Studies Schloß‐Wolfsbrunnenweg Heidelberg 69118 Germany; ^4^ Biosciences College of Life and Environmental Sciences University of Exeter Geoffrey Pope Building, Stocker Road Exeter EX4 4QD UK; ^5^ Sorbonne Université & CNRS UMR 7144 (AD2M) Station Biologique de Roscoff Place Georges Teissier Roscoff 29680 France; ^6^ Department of Ecology University of Kaiserslautern Kaiserslautern Germany; ^7^ CIRAD UMR LSTM Montpellier France; ^8^ Ecologie Systématique Evolution CNRS Université Paris‐Sud AgroParisTech Université Paris‐Saclay Orsay France; ^9^ School of Biological Sciences University of Bristol 24 Tyndall Avenue Bristol BS8 1TQ UK

**Keywords:** *Mitosporidium*, *Nucleophaga*, *Paramicrosporidium*, *Rozella*, Rozellida, Rozellomycota

## Abstract

Some protists with microsporidian‐like cell biological characters, including *Mitosporidium*,* Paramicrosporidium*, and *Nucleophaga*, have SSU rRNA gene sequences that are much less divergent than canonical Microsporidia. We analysed the phylogenetic placement and environmental diversity of microsporidian‐like lineages that group near the base of the fungal radiation and show that they group in a clade with metchnikovellids and canonical microsporidians, to the exclusion of the clade including *Rozella*, in line with what is currently known of their morphology and cell biology. These results show that the phylogenetic scope of Microsporidia has been greatly underestimated. We propose that much of the lineage diversity previously thought to be cryptomycotan/rozellid is actually microsporidian, offering new insights into the evolution of the highly specialized parasitism of canonical Microsporidia. This insight has important implications for our understanding of opisthokont evolution and ecology, and is important for accurate interpretation of environmental diversity. Our analyses also demonstrate that many opisthosporidian (aphelid+rozellid+microsporidian) SSU V4 OTUs from Neotropical forest soils group with the short‐branching Microsporidia, consistent with the abundance of their protist and arthropod hosts in soils. This novel diversity of Microsporidia provides a unique opportunity to investigate the evolutionary origins of a highly specialized clade of major animal parasites.

MICROSPORIDIA are conventionally considered as highly derived parasitic protists sister to *Rozella* or diverging as the next branch below the fungi (James et al. 2013). Microsporidia, Balbiani 1882 display a suite of distinctive cell biological characters related to their obligate parasitic lifestyle, including a characteristic spore‐extrusion apparatus (represented most conspicuously by the polar filament and its terminal anchoring disc) (Franzen 2004; Vávra and Lukeš 2013), unwalled intracellular trophic (meront) stages, and multiwalled spores produced by merogony or other forms of proliferation (Vávra and Larsson 1999). Microsporidia lack canonical Golgi apparatus (Beznoussenko et al. 2007; Vávra and Larsson 1999; Vávra and Lukeš 2013) and their mitochondria have been highly reduced to mitosomes (reviewed in Dean et al. 2016). These mitosomes are unable to generate their own ATP through oxidative phosphorylation, requiring energy to be imported from the host via nucleotide transporters. Microsporidia also lack flagella and an apparent capacity for phagocytosis. The known diversity of Microsporidia is large, comprising approximately 1,300 described species (Vávra and Lukeš 2013), forming a long‐branched clade (hereafter referred to as LB‐Microsporidia) in SSU rRNA and multigene phylogenies (James et al. 2013). Metchnikovellids (e.g. *Amphiamblys*,* Amphiacantha*) have traditionally been referred to as atypical, “primitive” microsporidians, but share many characters with LB‐Microsporidia, and were recently shown to branch as sisters to them (Mikhailov et al. 2017).

Microsporidia are known primarily as parasites of invertebrates and vertebrates (including humans), but are also known as endosymbionts of ciliates (Fokin 2012; Fokin et al. 2008), and hyperparasites in protists: metchnikovellids are parasites of gregarines, protistan gut symbionts of many invertebrates, and *Hyperspora aquatica* is a hyperparasite of the paramyxid *Marteilia cochillia*, a serious pathogen of European cockles (Stentiford et al. 2017). *Rozella* species are zoosporic biotrophic parasites of oomycetes, chytrids, and Blastocladiomycota (Spatafora et al. 2017). Increased attention has recently been given to a large diversity of lineages shown by phylogenetic analyses including environmental sequences to be related to Microsporidia, rozellids, and aphelids. The first to highlight this diversity were Lara et al. (2010) and Jones et al. (2011), who showed a large diversity of environmental sequences which, in the absence of microsporidian sequences, group with *Rozella* in phylogenetic trees. These have been referred to as Rozellida (Fig. 1 in Lara et al. 2010), Rozellomycota (Corsaro et al. 2014a,b), and Cryptomycota (Fig. 1 in Jones et al. 2011).

A few other sequences branching between rozellids and LB‐microsporidia represent microsporidia‐like protists which have been morphologically characterized: *Nucleophaga* (Corsaro et al. 2014a, 2016), *Paramicrosporidium* (originally described as a microsporidian; Michel et al. 2000, 2009), and *Mitosporidium* (Haag et al. 2014).

These three genera clearly share some features with classical LB‐Microsporidia, including forms of polar filaments (not necessarily functional as extrusion apparatus), unwalled intracellular meront stages, and nonflagellated spores; but in other respects, they are dissimilar, *Nucleophaga* and *Paramicrosporidium* being the least structurally similar to LB‐Microsporidia. *Mitosporidium*, sometimes referred to as the earliest branching microsporidian (Mikhailov et al. 2017; Quandt et al. 2017), has a mitochondrion, albeit lacking Complex I of the oxidative phosphorylation pathway. *Paramicrosporidium* has a canonical fungal mitochondrial genome, and shares more gene content with distantly related fungi than with its closest relatives (Quandt et al. 2017). *Mitosporidium* and *Nucleophaga* have possibly nonhomologous finger‐like extensions in naked intranuclear trophic stages similar to those of *Rozella* (Corsaro et al. 2014b; Haag et al. 2014).

Most published phylogenetic analyses that include Crypto/Rozellomycota/rozellid environmental sequences do not also include LB‐Microsporidia. Those that do (Corsaro et al. 2016 (Fig. 3); Tedersoo et al. 2017) suggest that *Rozella* is sister to a highly diverse clade comprising LB‐Microsporidia, the microsporidian‐like protists described above, and a large diversity of uncharacterized environmental sequences. In this study we investigate these relationships further, integrating morphological, phylogenetic, and sequence diversity data, to determine the phylogenetic and taxonomic boundaries of microsporidia and their immediate relatives.

## Materials and Methods

The nr nucleotide GenBank database was blastn searched using seed SSU rRNA gene sequences of characterized microsporidia‐like protists, metchnikovellids, deeply branching LB‐Microsporidia (clades 2 and 3 Stentiford et al. 2017), and the phylogenetic diversity of “Cryptomycota” and aphelids in Karpov et al. (2014). The top 50 matches for each sequence were downloaded, aligned with mafft e‐ins‐i (Katoh and Standley 2013), deduplicated, and a preliminary tree constructed on the basis of which the number of closely related sequences was reduced, retaining the longest possible sequences. The shorted branched LB‐Microsporidia *Janacekia*,* Trichonosema*, and *Bacillidium* were selected to represent LB‐Microsporidia in order to reduce the possibility of phylogenetic artefacts caused by LBA. Published phylogenies (e.g. Stentiford et al. 2017) show that these form a very robust monophyletic group with all other LB‐Microsporidia, so it is reasonable to use them as a proxy for the whole group in this study. A Bayesian phylogeny was inferred under the CAT+GTR+Gamma(4) model in PhyloBayes‐MPI 1.7 (Lartillot et al. 2013). Convergence among four MCMC chains was assessed by comparing the discrepancies in bipartition frequencies and in a range of continuous model parameters, along with the effective sample sizes of the continuous parameters. A consensus tree was built once all discrepancies were < 0.1, with sample sizes > 100. A maximum likelihood phylogeny was estimated under the GTR+Gamma(4)+F model in IQ‐Tree (Nguyen et al. 2015), with 200 traditional nonparametric bootstraps. The distribution of microsporidian characters (Table 1) was constructed from the literature. OTUs assigned to any of “Opisthosporidia” (Karpov et al. 2014); “Cryptomycota” (Jones et al. 2011), “Holomycota” (Liu et al. 2009), and “Microsporidia” and otherwise unassigned “fungi” by the taxonomic assignment algorithms of each study were extracted from environmental amplicon sequencing data of tropical forest soils clustered into OTUs by Swarm v2.1.5 (Mahé et al. 2015) and European coastal water and sediment samples (Logares et al. 2014). We inferred a maximum likelihood tree from our reference database using RaxML v8.2.8 (Stamatakis 2014). The OTUs were then aligned to the reference database using PaPaRa (Berger and Stamatakis 2011) and placed on the tree by RaxML‐EPA (Berger et al. 2011). The distribution of placements (Fig. 2, 3) was created with Genesis ( http://genesis-lib.org/) and visualized with FigTree ( http://tree.bio.ed.ac.uk/software/figtree/). Based on the 49 flagellar toolkit proteins assembled for a previous study (Torruella et al. 2015), we searched by BLAST in available early‐branching microsporidian predicted protein sets from genomic data, in *Mitosporidium daphniae* (Haag et al. 2014), *Paramicrosporidium* (Quandt et al. 2017), and *Amphiamblys* sp. (Mikhailov et al. 2017), using an e‐value threshold of 1e‐10 and manual scrutiny.

**Table 1:** Molecular and genomic characteristics of Microsporidia and their relatives. Two long‐branch microsporidia infecting nematodes (*Nematocida parisii*) and vertebrates (*Encephalitozoon cuniculi*) and *Rozella* (Rozellida)) are shown in comparison. Dashes represent unavailable data. ✓/x indicate presence/absence of a character; where uncertain these are shown in parentheses. Note that, following ancestral reduction, some LB‐Microsporidia have experienced independent secondary expansions in genome size (e.g. up to 51.3 Mb in *Edhazardia aedis* Desjardins et al. 2015)), but the additional material largely comprises noncoding and repetitive sequences, without a concomitant increase in cellular or metabolic complexity. The polar filament was reported as absent in *Nucleophaga amoebae*, although a form of it is present in the very closely related *N. terricolae*. It is possible that it has been lost in. *N. amoebae* or was simply not seen in the cells investigated by Corsaro et al. (2014b)

	Flagellate stage	Genome size (Mb)	Gene density genes/kbp	Number of protein coding genes	ATP/DP translocases (HGT)	Mitochondrial genome	Electron transport chain	Polar filament			Spore	
								Regular	Atypical	Absent	Exo+endospore	Posterior vacuole
*Encephalitozoon cuniculi* (LB‐Microsporidia)^1,2^	x	2.9	0.81	1,997	✓	x	x	✓			✓	✓
*Nematocida parisii* ERTm1 (LB‐Microsporidia)^3,4^	x	4.1	0.65	2,661	✓	x	x	✓			✓	✓
*Amphiamblys* sp. WSBS 2006 (metchnikovellid)^5^	x	5–7	0.66	2,529 (nr)	x^a^	x	x	‐	‐	‐	‐	‐
*Amphiamblys capitellides* (metchnikovellid)^6^	x	‐	‐	‐	‐	‐	‐		✓		✓	x
*Mitosporidium daphniae* (SB‐Microsporidia)^7^	x	5.64	0.585	3,300	x	✓	Partial	✓			✓	x
*Paramicrosporidium saccamoebae* (SB‐Microsporidia)^8,9^	x	7.3	‐	3,750	x	✓	✓		✓		✓	x
LKM11‐01; CM1.1 (SB‐Microsporidia)^10^	✓	‐	‐	‐	‐	‐	‐	‐	‐	‐	‐	‐
*Nucleophaga terricolae* (SB‐Microsporidia)^11,12^	x	‐	‐	‐	‐	‐	‐		✓		✓	x
*Nucleophaga amoebae* (SB‐Microsporidia)^13^	x	‐	‐	‐	‐	‐	‐			✓?^b^	c	x
*Rozella allomycis* (rozellid)^14^	✓	11.9	0.535	6,350	✓	✓	Partial			✓	x	x

nr, nonredundant. 1. Katinka et al. (2001), 2. Karpov et al. (2014), 3. Cuomo et al. (2012), 4. Troemel et al. (2008), 5. Mikhailov et al. (2017), 6. Larsson and Koie (2006), 7. Haag et al. (2014), 8. Corsaro et al. (2014a), 9. Quandt et al. (2017), 10. Jones et al. (2011), 11. Corsaro et al. (2016), 12. Michel et al. (2012), 13. Corsaro et al. (2014b), 14. James et al. (2013), 15. Letcher and Powell (2005), and 16. Ajello (1948).

a Incomplete genome (estimated 90% complete).

b Undetected or potential loss.

c Two‐layered cell wall.

## Results and Discussion

### An expanded Microsporidia

**Figure 1:**
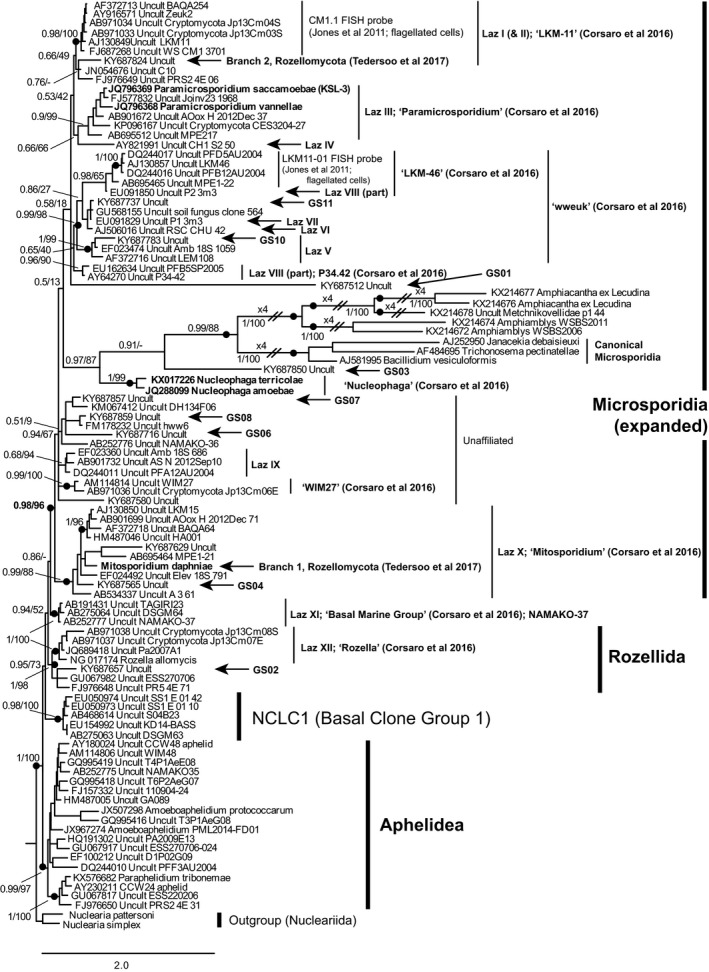
Phylogenetic relationships among canonical, long‐branching (LB) Microsporidia, metchnikovellids, Rozellida, and a diversity of related short‐branching lineages. LB‐Microsporidia form a clade with a diversity of short‐branching lineages (SB‐Microsporidia) that share key cell biological characters defining the microsporidian clade. Lineages that have been labelled in other studies are labelled: GS *xx* from Tedersoo et al. (2017), Laz *x* from Lazarus and James (2015); others as marked. The extent of the expanded Microsporidia is shown by the bracket on the far right. The tree was inferred under the CAT+GTR model in PhyloBayes‐MPI, on a final alignment of 1729 sites from the SSU rRNA gene. Black blobs indicate support values of > 0.96 BPP and > 95% ML bootstrap (actual values also shown). Branch lengths are proportional to the expected number of substitutions per site, as denoted by the scale bar.

We constructed an SSU rRNA gene sequence alignment including a comprehensive selection of the microsporidian‐like protists and their related environmental sequences, metchnikovellids, and representatives of short‐branch LB‐Microsporidia (see Methods), aphelids, rozellids, and related unclassified groups. Maximum Likelihood and Bayesian phylogenetic analyses (Fig. 1) showed that the expected metchnikovellid+LB‐Microsporidia clade emerges from within a diverse and well‐supported (Bayesian posterior probability (BPP) = 0.98; Maximum Likelihood bootstrap 96%) clade including *Paramicrosporidium*,* Nucleophaga*,* Mitosporidium*, LKM‐11, and many other environmental sequences referred to as rozello‐/cryptomycotans. *Rozella* formed a clade with high support with a relatively small number of environmental sequences.

**Figure 2:**
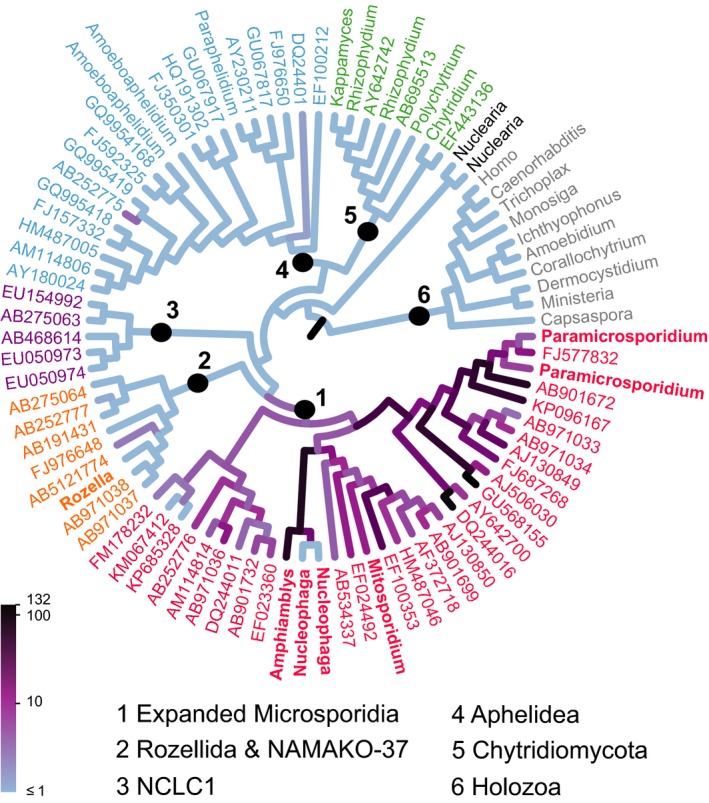
Evolutionary placement of tropical soil microsporidian OTUs on the opisthosporidian reference tree. The branch colours correspond to the distribution of OTU placement: The darker a branch, the more OTUs are placed on it. The OTU frequency scaling is logarithmic due to the large range of placement density. The branch labels for Microsporidia are red (characterized taxa in bold), rozellids orange, NCLC1 purple, aphelids blue, chytrids green, and the holozoan outgroup grey. Nucleariids are the sister clade to Fungi+Opisthosporidia.

It is well recognized that SSU rRNA gene data alone are unable to resolve deep phylogenetic relationships. However, although not a substitute for multigene data, the high taxon sampling afforded by SSU provides phylogenetic information currently inaccessible for phylogenomic analyses (Berney et al. 2004; Cavalier‐Smith 2004). We calculated a range of trees of varying taxon samplings (e.g. Fig. 2; other data not shown), and although the branching order within the clade was not consistent between them (although many subclades were consistently recovered), the microsporidian clade as marked on Fig. 1 was invariably and strongly recovered.

These analyses provide additional evidence for an expanded and strongly supported microsporidian clade, including all of the LB‐Microsporidia, (metchnikovellids, the “microsporidian‐like” protists discussed above, and almost all of the environmental “crypto/rozellomycotan” diversity indicated in Lara et al. 2010; Jones et al. 2011; Corsaro et al. 2014a,b, 2016; Karpov et al. 2014; Lazarus and James 2015; Tedersoo et al. 2017), but excluding rozellids (=*Rozella*), NAMAKO‐37, and NCLC1 (Basal Clone Group 1). We therefore propose that this clade including the large diversity of environmental sequences, are all actually microsporidia, and we refer to them (excluding LB‐Microsporidia) here as short‐branched Microsporidia (SB‐Microsporidia). For the purposes of this study we also exclude metchnikovellids from the definition of SB‐Microsporidia as sequenced metchnikovellids have significantly longer branches than the SB‐Microsporidia shown on Fig. 2. However, it is likely that as more related lineages are discovered a more gradual continuum of branch lengths between SB‐Microsporidia such as *Nucleophaga* (Fig. 2), metchnikovellids, and LB‐Microsporidia will be revealed.

This “expanded” Microsporidia concept is consistent with the original descriptions of *Paramicrosporidium* (Michel et al. 2000, 2009) as “microsporidian” or “microsporidian‐like”, and *Mitosporidium*, which exhibits merogony and a coiled polar filament, the latter exclusive to Microsporidia, being “profoundly morphologically similar to Microsporidia” (Haag et al. 2014). This phylogenetically broader circumscription of Microsporidia is morphologically distinct because all characterized lineages in the microsporidian clade possess the key morphological features of Microsporidia: spores with multilayered cell walls containing polar filament apparatus (Richards et al. 2017; Vávra and Lukeš 2013), and merogony, whereas *Rozella*, aphelids, and fungi do not possess these characters.

### Morphological vs. genomic evolution in Microsporidia

Corresponding morphological and genomic datasets are available for only a very small proportion of lineages representing SB‐Microsporidia and metchnikovellids. Until very recently this applied to only *Mitosporidium* (Haag et al. 2014) and *Amphiamblys* (Mikhailov et al. 2017), but the addition of nuclear and mitochondrial genomes of *Paramicrosporidium saccamoebae* (Quandt et al. 2017) have provided several intriguing new perspectives on microsporidian evolution. It is increasingly apparent that, although all SB‐microsporidia exhibit microsporidian‐defining morphological characters, their genomic evolution appears far more mosaic (Quandt et al. 2017); Table 1. *Rozella* and LB‐Microsporidia both have horizontally acquired *Rickettsia*‐like NTT ATP/ADP transporters, but metchnikovellids, *Mitosporidium* and *Paramicrosporidium* do not (Table 1). LB‐Microsporidia lack mitochondrial genomes, which are present in *Mitosporidium* and *Paramicrosporidium*. The mitochondrial genomes of *Mitosporidium* and *Rozella* lack Complex I of the oxidative phosphorylation pathway, are degenerate, and AT‐rich, but that of *Paramicrosporidium* includes all genes of that pathway typically found in fungi; in fact, the total gene complement of both *Paramicrosporidium* and *Mitosporidium* have more in common with fungi than with its closest relatives (Haag et al. 2014; Quandt et al. 2017). The very fast rates of sequence evolution (and so long branches in the phylogeny) observed for LB‐Microsporidia correlate with extensive cellular and genomic reduction, including strong mutational bias to AT and the loss of some DNA replication and repair genes that, in other eukaryotes, help to promote genome stability (Williams et al. 2016). While genome data are currently very sparse, the slower evolutionary rates observed for the 18S genes of SB‐Microsporidia suggest that, while these organisms are also parasites, reductive evolution has not proceeded to the same extreme degree as in the LB clade. Another instance of potentially horizontally acquired genes (the distribution of which may understandably not correlate with phylogeny) are thymidine kinases found in *Rozella* and LB‐Microsporidia, but not *Paramicrosporidium* (Alexander et al. 2016; Quandt et al. 2017). *Paramicrosporidium* possesses a full set of meiosis genes and significant evidence for diploidy, more similar to LB‐Microsporidia than is known to be the case for other SB‐Microsporidia. In summary, as Quandt et al. (2017) note, “shared gene content is clearly not correlated with evolutionary relationships”; instead, gene content evolution within the microsporidian clade depicted in Fig. 1 appears to be characterized by repeated, lineage‐specific gene losses rather than a stepwise trend towards genome reduction in LB‐Microsporidia.

### Flagella and polar filament evolution in Microsporidia

While rozellids have a lifecycle of alternating zoosporic and nonflagellate trophic stages, flagellate (zoosporic) stages are unknown for any lineages in the expanded microsporidian clade, with two intriguing exceptions. Jones et al. (2011) showed that members of two SB‐microsporidia lineages (Laz I and LKM‐46, indicated on Fig. 1) have zoosporic stages without chitin in their cell walls. Assuming the cells reconstructed from those FISH experiments represent the branches indicated on Fig. 1 and were not false‐positive FISH signals, this shows that some SB‐Microsporidia do possess flagella at some stage of their life cycle. In this study, we used BLASTP to search for homologs of the specialized epsilon and delta tubulins, intraflagellar transport system, or flagellar‐specific motor molecules in the genomic datasets for the putatively earlier‐diverging *Mitosporidium* (or the metchnikovellid *Amphiamblys* sp.), but were unable to find any significant hits (E < 1e‐10). We were also unable to find significant hits to most of the proteins associated with flagellar structure and function detected in *Paramicrosporidium* by Quandt et al. (2017). These analyses suggest that none of these lineages has a cryptic flagellum that might have been missed by microscopy. However, the branching position of the lineages targeted by FISH in Jones et al. (2011) is unresolved within the microsporidian clade. If these lineages are actually more deeply branching than other characterized SB‐Microsporidia then it is possible that the flagellar apparatus in microsporidia was lost early in the diversification of the clade. If this is the case, it would be very interesting to know whether this pre‐dated or overlapped with the evolution of the polar filament apparatus, and whether the latter arose at the origin of the microsporidian clade. Alternatively, it is possible that only some SB‐Microsporidia lost their flagella apparatus (and associated genes) and that those lineages represented in Jones et al. (2011) are exceptions to the generality suggested by the other characterized lineages in this part of the tree. In that case the use of FISH enabled detection of life‐stages and lineages that have so far eluded cell isolation‐based methods of investigation. Screening genomic data alone may not provide all such information: even though *Paramicrosporidium* has an obvious polar filament, polar filament proteins (PFPs) known from LB‐Microsporidia were not found in the *Paramicrosporidium* or *Mitosporidium* genomes (Haag et al. 2014; Quandt et al. 2017), suggesting rapid evolution of PFPs in the microsporidian clade, earlier forms of these proteins being too dissimilar to their highly derived homologs to be detectable by gene similarity searches. This observation is supported by the low number (589–664; 24–27%) of orthologous genes shared between *Paramicrosporidium* and all sequenced LB‐Microsporidia. Haag et al. (2014) found only four orthologs shared between *Mitosporidium* and LB‐Microsporidia, but not with other fungi.

The small number of genomic comparisons currently possible between members of Opisthosporidia shows a mosaic evolution, at least partly mediated by horizontal gene transfer, independent gene losses, and perhaps multiple transitions to parasitism. This is a fascinating situation, worthy of intense study, but does not detract from a simple and robust classification as proposed here. As more lineages are detected and characterized, this heterogeneity is set to increase. By adopting a phylogenetically driven, character‐based classification structure based on the monophyly of an expanded microsporidian clade, our rationalization provides a clearer set of hypotheses on which to base future studies by pinpointing the origin of the microsporidian radiation, the relation to which genomic and cellular characters can be ascertained.

### Classification of Microsporidia and their relatives

Taxonomic circumscriptions of Rozellomycota and, particularly, Cryptomycota, vary significantly (see Berbee et al. 2017; Spatafora et al. 2017; Richards et al. 2017; and examples cited throughout this study). Some authors include aphelids within Cryptomycota (e.g. Letcher et al. 2013), but more normally Crypto/Rozellomycota are used to encompass *Paramicrosporidium*,* Mitosporidium*,* Nucleophaga*, the lineages detected by FISH in Jones et al. (2011), the strongly supported clade containing *Rozella*, and the large diversity of environmental sequences branching around and among these lineages. This is the most frequently used classification, informally described by Letcher et al. (2018) and shown in Jones et al. (2011), Lazarus and James (2015), and (excluding *Mitosporidium*, which is classified as microsporidian) Quandt et al. (2017).

We suggest that the frequently referred to “paraphyly of Rozello/cryptomycota” is both misleading and avoidable. The defining morphological characters of microsporidia are spores with multilayered cell walls containing polar filament apparatus homologs (not necessarily functioning in extrusion). The phylogenetic distribution of these characters is coincident with the clade containing only SB‐ and LB‐Microsporidia, which is recovered by both SSU and multigene phylogenetic analyses. Therefore, all members of that clade are in fact Microsporidia, and there is no need for them to be regarded as belonging to a paraphyletic group. The closest known relatives of microsporidia, again according to SSU (Fig. 1) and multigene phylogenies, are rozellids, which are restricted to a robustly supported clade including the genus *Rozella*. *Rozella* shares some microsporidian‐like features with the more distantly related LB‐Microsporidia (e.g. horizontally acquired *Rickettsia*‐like NTT ATP/ADP transporters, degenerate, and AT‐rich mitochondrial genomes lacking Complex I of the oxidative phosphorylation pathway, thymidine kinases), but not with all of their shorter‐branch relatives, and are highly morphologically distinct.

What then are Cryptomycota (Jones et al. 2011) and Rozellomycota (Corsaro et al. 2014a)? On the basis that they refer to the same assemblage of lineages (which is usually the case) two names are unnecessary and confusing. The purpose of this study is neither to decide between them nor suggest an alternative, nor even to invalidate them. However, to be consistent with recent usage this label could be applied to an uncharacterized monophyletic group branching somewhere in the opisthosporidian clade, excluding aphelids, the expanded microsporidia, or the clade including *Rozella*. The latter already has order or class status, according to different authorities (Lara et al. 2010; Ruggiero et al. 2015). Figure 1 indicates the diversity revealed by general eukaryote‐wide environmental sequencing studies, including those clades coded and labelled by Corsaro et al. (2016), Tedersoo et al. (2017), and the diversity detected by the targeted PCR approach of Lazarus and James (2015). Of this very substantial environmental diversity, only two groups within Opisthosporidia do not branch within the expanded microsporidian, rozellid, or aphelid clades: (1) the NAMAKO‐37 clade (which in Corsaro et al. (2016) branches outside of the microsporidian clade before the divergence of *Rozella*, in Jones et al. (2011) is sister to *Rozella*, and in Fig. 1 of this study [under the best‐fitting CAT+GTR phylogenetic model], and in Lazarus and James (2015) is sister to microsporidia), and (2) NCLC1 (Basal Clone Group I), which in Fig. 1 branches between NAMAKO‐37 and aphelids, but whose actual branching position is unresolved (Richards et al. 2015, 2017). On the basis of their phylogenetic position and existing provenance data we suggest that the last two groups are zoosporic parasites of marine microbial eukaryotes.

We infer that the “unaffiliated” group (Fig. 1) is microsporidian (and therefore not cryptomycotan) based on tree topology and bipartition support. These cells could provide key insights into early microsporidian evolution. We hypothesize that they possess mitochondria, and possibly flagellar structures or/and simpler cell extrusion apparatus than in other SB‐Microsporidia. The NAMAKO‐37 clade cannot be classified until more data are available. There is negligible support for their being microsporidian or belonging to any other recognized group, so the label “Cryptomycota” could be used for this monophyletic lineage, at least for the time being. However, the message of this article remains unchanged whether the whole Rozellida + Microsporidia clade is referred to as Rozello‐ or Cryptomycota or whether these names fall out of use, what the relationships between aphelids, rozellids, and microsporidia actually are, and where the boundary between fungi and other protists is. Crucially, acknowledging an expanded Microsporidia as an evolutionarily and morphologically coherent unit both unambiguously clarifies the taxonomy/classification of this very interesting clade in the eukaryote tree, and provides a clear framework for future research.

### High diversity of SB‐Microsporidia in neotropical soils

An important consequence of our analyses is that the taxonomic affiliations of large‐scale SSU rRNA amplicon sequencing studies must now be revisited. Most of the sequences annotated as “crypto/rozellomycota” in taxonomically curated databases such as SILVA and PR^2^ (Guillou et al. 2013) are likely to be SB‐Microsporidia. The rozellid annotation should be restricted to the clade indicated on Fig. 1. This is not purely a matter of classification: Microsporidia and rozellids are each monophyletic and distinct in terms of biology and ecology, but are currently conflated in the major sequence databases. Clarifying the composition of the two clades will enable much more accurate and high‐resolution analyses, and interpretation of microsporidian diversity and function.

To demonstrate the particular relevance of this more inclusive definition of microsporidia to the annotation and interpretation of environmental sequencing studies, we reevaluated a recent SSU rRNA high‐throughput sequencing study of three Neotropical rainforest soils (Mahé et al. 2017), using the Evolutionary Placement Algorithm as implemented in RAxML (Stamatakis 2014) to place operational taxonomic units (OTUs) previously annotated as Opisthosporidia, crypto/rozellomycota, or unassigned fungi. We similarly analysed an OTU dataset from European coastal water and sediment samples (BioMarKs: Logares et al. 2014; Massana et al. 2015).

**Figure 3:**
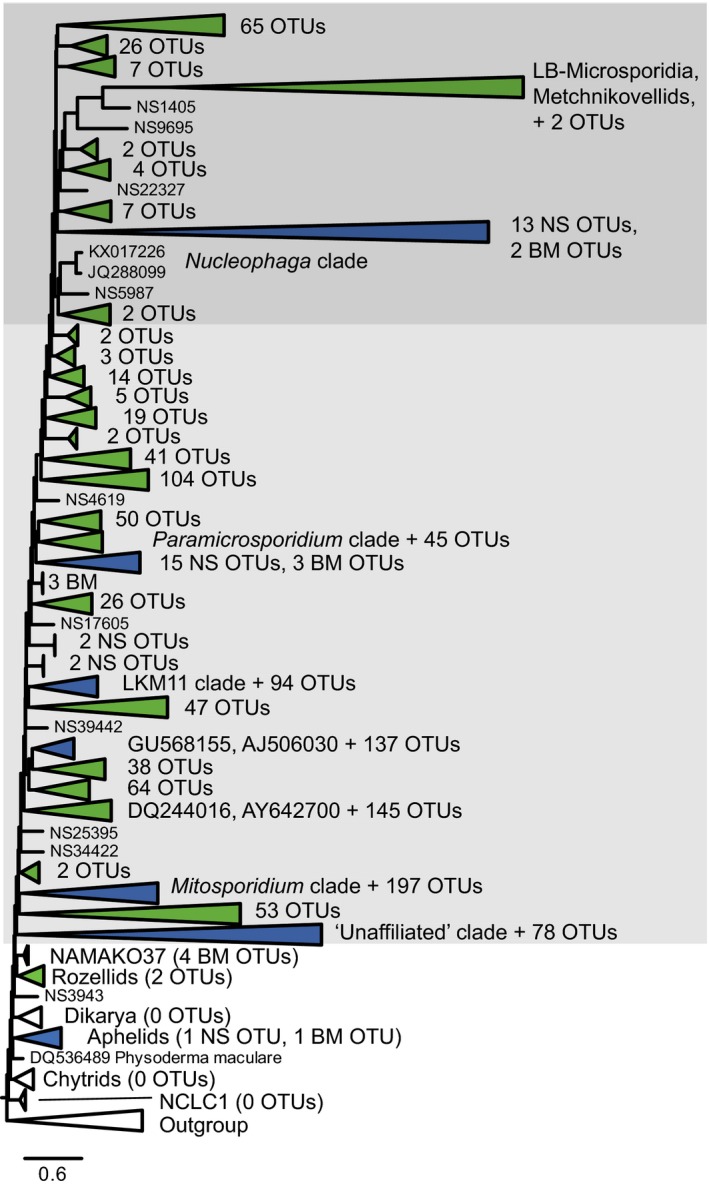
Maximum Likelihood phylogeny of SSU rRNA gene V4 region OTUs from tropical soil and European marine (Biomarks) samples. The OTUs are aligned to reference sequence dataset of long‐ and short‐branch Microsporidia, metchnikovellids, rozellids, other clades of environmental sequences, and representative aphelids and fungi with a holozoan outgroup. Light and dark grey shading indicates extent of expanded Microsporidia; dark grey box encloses all members of the subclade including long‐branch Microsporidia. Green triangles (collapsed clades) represent OTUs from Neotropical soil samples only, ‘BM’ from Biomarks only, and blue from both.

Of the 1,279 candidate tropical soils OTUs, 94% clustered within the microsporidian clade (Fig. 2, 3). Figure 3 shows the branching positions of clades of OTUs relative to the reference sequences. OTUs clustering with the metchnikovellid branch are putatively parasites of gregarines. Others grouping nearer *Nucleophaga*, at the base of the branch leading to metchnikovellids and LB‐Microsporidia, may be parasites of amoebae or/and other protists (perhaps including hyperparasites). Mahé et al. (2017) show that both gregarines and Amoebozoa are highly diverse in their Neotropical soil samples. However, until more lineages are found in nature we will be unable to conclusively determine whether microsporidian diversity branching nearer *Paramicrosporidium* or *Mitosporidium* are parasites of protists and arthropods respectively. Although there were far fewer opisthosporidian OTUs in the BioMarKs data, the majority of these also branched within the microsporidian clade (Fig. 3). In both datasets a small proportion of OTUs was placed in the rozellid clade (two from soil), and the NAMAKO‐37 clade (four from BioMarKs), consistent with the apparently limited diversity of these clades compared to Microsporidia.

LB‐Microsporidia are predominantly parasites of animals, but *Nucleophaga* and *Paramicrosporidium* are parasites of protists (amoebozoan hosts are only known so far, which are relatively scarce in marine habitats). Metchnikovellids are parasites of gregarines (protistan gut parasites of a wide range of invertebrates; Desportes and Schrével 2013). *Mitosporidium* is a parasite of *Daphnia* (Haag et al. 2014), and perhaps other lineages in the *Mitosporidium* clade are also parasites of arthropods. SB‐Microsporidia therefore appear to occupy a broad and little understood set of niches. The phylogenetic distribution of characters typically associated with LB‐Microsporidia may be determined at least as much by host‐specific adaptation as phylogenetic relatedness. For instance, shorter/reduced polar filaments may be more characteristic of Microsporidia infecting protists than invertebrates. On the other hand, if at least in some cases cell host invasion is mediated by host phagocytosis, the length/complexity of the polar filament may not always be directly related to the physical barriers it must cross to invade the host (Franzen 2004).

## Conclusions

We analysed the phylogenetic placement and environmental diversity of microsporidian‐like lineages that group near the base of the fungal radiation. These lineages form a monophyletic group including canonical Microsporidia and metchnikovellids, but excluding a strongly supported rozellid clade. This topology is concordant with the phylogenetic distribution of defining microsporidian cell characters, but not shared gene content across Opisthosporidia and fungi. The genetic diversity of Microsporidia is far higher than previously realized, and includes the SB‐microsporidian taxa *Mitosporidium*,* Paramicrosporidium*, and *Nucelophaga*, and many uncharacterized environmental sequence types. The concept of Rozellomycota/Cryptomycota requires revision to avoid encompassing lineages that are actually microsporidian. Our analyses suggest hypotheses for investigations into the relative timings of acquisition of the polar filament apparatus and loss of flagella, key microsporidian characteristics. We show that this revised classification has major implications for our understanding of microsporidian diversity as inferred from environmental sequencing surveys. The large diversity and abundance of SB‐Microsporidia offer unique opportunities to study the evolution of the highly specialized cells and genomes of canonical Microsporidia, and in particular their propensity for direct parasitism of animals.
